# Agroinfiltration technique for elucidating the functions of strawberry genes in *Fragaria vesca*

**DOI:** 10.1038/s41598-025-08344-0

**Published:** 2025-07-01

**Authors:** Chonprakun Thagun, Yutaka Kodama

**Affiliations:** Center for Bioscience Research and Education (C-Bio), Utsunomiya University, 350 Mine-Machi, Utsunomiya, Tochigi, 321-8505 Japan

**Keywords:** *Agrobacterium tumefaciens*, Agroinfiltration, Genetic engineering, Promoter analysis, Strawberry, Transactivation assay, Biotechnology, Genetics, Molecular biology, Plant sciences

## Abstract

Unraveling gene function is crucial for enhancing quality traits in commercially cultivated strawberry (*Fragaria* × *ananassa*) through biotechnology. However, such analysis is hindered by the complexity of the octoploid genome (2*n* = 8*x* = 56) of *F.* × *ananassa*. In this study, we leveraged the ancestral wild strawberry species *Fragaria vesca* as an experimental model and developed an *Agrobacterium tumefaciens*–based leaf infiltration (agroinfiltration) technique to elucidate the functions of genes and regulatory sequences in strawberry. We transiently expressed genes encoding transcriptional regulators that govern floral organ development in *F. vesca* leaf cells, allowing us to investigate the underlying regulatory mechanisms through *in planta* gene expression and transactivation assays. Our agroinfiltration method serves as a practical tool for studying the activities of genes controlling economically important traits in strawberry.

**Supplementary Information:**

The online version contains supplementary material available at 10.1038/s41598-025-08344-0.

## Introduction

Commercially cultivated strawberry (*Fragaria* × *ananassa*), a member of the Rosaceae family, arose through genetic hybridization between two parental strawberries, *F. virginiana* and *F. chiloensis* ^1^. In addition to their delicious aroma and taste, strawberries are an excellent source of essential bioactive compounds including vitamins and antioxidants that provide numerous health benefits ^2^. However, various factors negatively affect the nutritional quality and productivity of globally cultivated strawberry, including fluctuating growth conditions (such as temperature, relative humidity, soil salinity, and light conditions) and biotic stress (including pathogens and insect infestation) ^1,2^. To overcome these challenges, it is essential to improve cultivation practices and expand our understanding of the genetic variation associated with abiotic and biotic stress responses in strawberry. To this end, it is crucial to identify the genes involved in agronomic traits and stress responses for basic research and the biotechnological improvement of quality traits in strawberry. However, *F.* × *ananassa* is of limited practical use in molecular genetic studies because it has a complex octoploid genome (2*n* = 8*x* = 56) and is only cultivated seasonally ^1^.

Transient expression is a rapid, cost-effective approach for analyzing plant gene functions. Methods such as *Agrobacterium tumefaciens* (syn. *Rhizobium radiobacter*)–mediated gene transfer through infiltration into plant tissues (agroinfiltration), protoplast transfection, particle bombardment, virus-induced gene silencing, and emerging nanotechnology-based delivery systems enable transient gene expression across various plant species ^3^. These transient expression systems are used for functional genomics, promoter analysis, analyzing protein–protein interactions, recombinant protein production, and testing CRISPR/Cas9-mediated edits in the genome. Model plants such as *Nicotiana benthamiana* are commonly employed in these assays due to their strong susceptibility to *Agrobacterium* and high rates of protein production ^4^. The advantages of transient expression include its speed, scalability, and reduced costs compared to stable transformation. Despite its limitations, such as the short expression duration and limited efficacy in some tissues and plant species, transient expression continues to play a crucial role in advancing basic plant research and biotechnology.

Agroinfiltration is a valuable technique for elucidating the functions of strawberry genes by enabling the transient expression of target genes or the silencing of specific genes in strawberry tissues ^5–7^. This method allows researchers to quickly assess the roles of genes in processes such as fruit development, ripening, disease resistance, and metabolic pathways, providing insights into their biological functions. The use of agroinfiltration to deliver genetic constructs into strawberry fruits is especially advantageous for studying the functions of genes and proteins in a cost-effective and time-efficient manner ^7^. However, agroinfiltration in strawberry fruit faces several challenges, including variability in infiltration efficiency due to the structural complexity of strawberry fruits; transient gene expression, which limits long-term studies; and potential interference from the plant’s immune responses to *Agrobacterium*. Leaf-based assays offer distinct advantages. Leaves are more accessible, allow for faster experimental execution, and typically yield more uniform and reproducible results. These benefits make leaf-based agroinfiltration a particularly efficient and practical option for transient gene expression studies in strawberries. Ongoing improvements in delivery methods and host compatibility holds promise for expanding the use of agroinfiltration for functional genomics studies of strawberry.

Wild strawberry (*Fragaria vesca*), an ancestor of commercially available hybrid strawberry, harbors genetic information comparable to that of cultivated strawberry (*F.* × *ananassa*) within its diploid genome (2*n* = 2*x* = 14) ^1,8^. The shorter growth period and fruiting cycle of *F. vesca*, along with its ability for vegetative propagation using stolons (runners) and its distinctive floral morphology, make *F. vesca* an ideal material for physiological studies of strawberry ^8,9^. Additionally, the availability of updated genome data for *F. vesca* facilitates the genomic analysis and genetic engineering of functionally relevant, economically important genes in *F.* × *ananassa* ^8,10^. These remarkable advantages have positioned *F. vesca* as a valuable model for functional genomics studies, shedding light on gene functions and regulatory networks associated with key traits in commercial strawberry.

Advancements in genetic transformation techniques for wild strawberry (*F. vesca*) have led to significant advances in studying gene function ^5–7^. Notably, the development of diverse transformation protocols highlights the great progress in manipulating the genome of this wild strawberry ^11,12^. While these advances increase the opportunity for studying gene functions, several challenges remain, such as the improvement of transformation efficiency and the optimization of regeneration protocols. These challenges must be addressed to fully unlock the potential of genetic transformation in *F. vesca* for strawberry improvement. Furthermore, a straightforward method for validating gene expression using transient expression must be established to facilitate subsequent analysis, including developing multi-omics approaches in strawberries.

In this study, we developed an *Agrobacterium*-based gene transfer protocol for transient gene expression analysis in wild strawberry (*F. vesca*). We successfully introduced transgene expression cassettes into intact leaf tissue via agroinfiltration. Using this technique, we performed functional studies of a transcription factor, including the regulatory sequences and promoter activities of its target genes within intact leaves of *F. vesca*. This transformation technique is a practical tool for investigating economically important traits in wild and commercially cultivated strawberry.

## Results

### *Agrobacterium*-mediated leaf infiltration

**Fig. 1:**
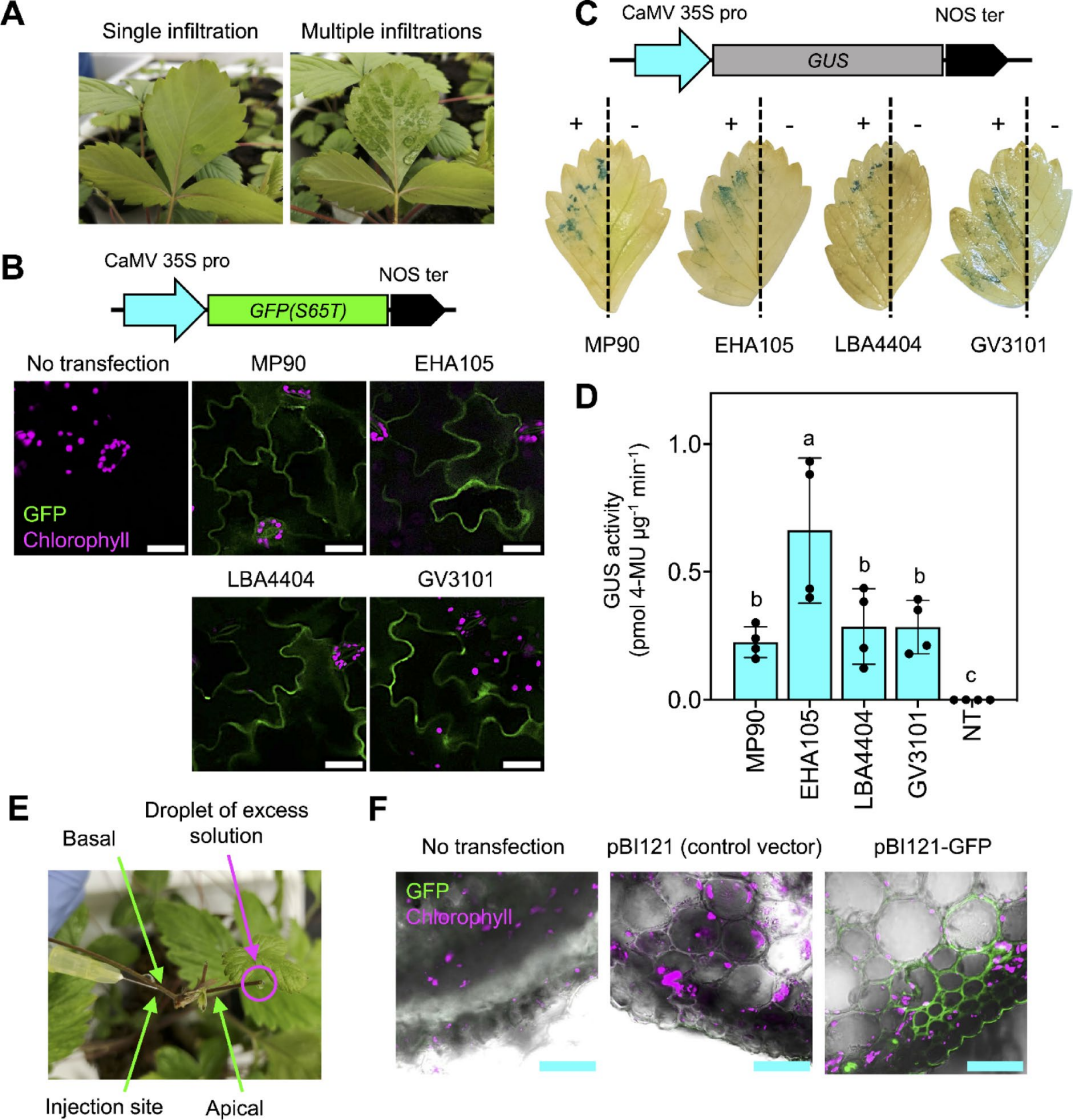
Transient expression of reporter genes in wild strawberry using *Agrobacterium*-mediated infiltration. (**A**) Agroinfiltration of a *F. vesca* leaf was initially conducted by infiltrating at a single spot (single infiltration, left panel) on the abaxial side of the leaf. The right panel shows a leaf infiltrated in multiple spots to ensure comprehensive coverage of the leaf surface. (**B**) Confocal laser-scanning microscopy images of *F. vesca* leaf cells transfected with the GFP expression cassette at 3 days after infiltration (DAI). CaMV 35S pro represents the strong constitutive Cauliflower mosaic virus (CaMV) 35S promoter. NOS ter indicates the terminator sequence of the *nopaline synthase* (*NOS*) gene. Scale bars represent 50 μm. (**C**) Spatial expression of the GUS reporter in agroinfiltrated *F. vesca* leaves at 3 DAI. *Agrobacterium* carrying the GUS expression cassette was infiltrated into one half of each *F. vesca* leaf (+), and the other half of the leaf was used as the non-infiltrated control (−). (**D**) Transient expression of the GUS reporter in agroinfiltrated *F. vesca* leaves at 3 DAI. *F. vesca* leaves were infiltrated with solutions containing different strains of *Agrobacterium* harboring pBI121, a binary vector containing the bacterial *β-glucuronidase* (*GUS*) gene. NT represents control leaf samples with no transfection. Error bars show standard deviations of the mean GUS activity in four agroinfiltrated leaves from two biologically independent experiments (*n* = 4). Different letters indicate statistically significant differences in mean GUS activity, as analyzed by one-way ANOVA with Tukey’s HSD test at *p* = 0.05. (**E**) *Agrobacterium* injection to introduce a gene expression cassette into vascular cells of wild strawberry runners. (**F**) Expression of the GFP reporter in an injected strawberry runner. CLSM was used to observe GFP expression in cross-sections of the apical ends at 3 DAI. Scale bars represent 200 μm.

We initially conducted agroinfiltration using fully expanded *F. vesca* leaves to optimize our transient gene expression technique (Fig. 1). Our attempt to infiltrate intact *F. vesca* leaves at a single location was ineffective for spreading the agroinfiltration solution over the entire leaf surface (Fig. 1A). Therefore, we repeatedly infiltrated the abaxial sides of *F. vesca* leaves at multiple spots to facilitate the dispersion of the agroinfiltration solution. This treatment greatly improved the distribution of agroinfiltration solution within leaf tissues (Fig. 1A).

*Agrobacterium* strains, such as GV3101, LBA4404, and EHA105, have demonstrated different efficiencies for introducing gene expression cassettes into strawberry ^13,14^. However, the transformation efficiencies of these strains in *F. vesca* tissues remained to be analyzed. First, we validated the expression of the green fluorescent protein (GFP) gene in plant cells following agroinfiltration using various strains of *Agrobacterium* (MP90, EHA105, LBA4404, and GV3101) harboring the GFP expression vector pBI121-GFP(S65T) (Fig. 1B). The expression of recombinant GFP in the transfected leaf cells was visualized using confocal laser-scanning microscopy (CLSM) at 3 days after infiltration (DAI). All *Agrobacterium* strains examined resulted in sufficient gene expression and the cytosolic localization of GFP within the transfected cells of *F. vesca* leaves (Fig. 1B).

We introduced agroinfiltration solutions carrying *Agrobacterium* cells harboring the *β-glucuronidase* (*GUS*) gene expression cassette into multiple areas of *F. vesca* leaves through leaf infiltration to evaluate the transformation efficiencies of different *Agrobacterium* strains (Fig. 1C). Subsequently, we conducted histochemical staining and enzymatic assays to assess the expression of the GUS reporter in transiently transfected leaves at 3 DAI (Fig. 1C and D). Histochemical staining revealed localized expression of the GUS reporter in the transformed leaf areas, in contrast to the non-infiltrated controls (Fig. 1C). We also detected a significant increase in GUS reporter activity in agroinfiltrated *F. vesca* leaves compared to samples without infiltration (Fig. 1D). Fluorescence microscopy of GFP signals and GUS activity assays suggested that the gene of interest can be efficiently expressed and analyzed across multiple areas of the abaxial leaf surface of *F. vesca* using our agroinfiltration technique. Among all *Agrobacterium* strains examined, infiltration with EHA105 led to the highest transient expression of the GUS reporter in *F. vesca* leaves (Fig. 1C and D). Therefore, we chose *Agrobacterium* strain EHA105 for subsequent analysis.

To investigate the potential use of *F. vesca* stolons (runners) as the plant material for *in planta* transformation of strawberry genes, we introduced a solution containing the fluorescent dye rhodamine B into the basal runner segments of daughter plants using syringe injection until a droplet of excess injected solution was observed at the tip of the apical runner (Fig. 1E). We assessed the translocation of rhodamine B dye within the runner at 30 min post injection using fluorescence microscopy. CLSM images revealed that rhodamine B spread from the injection site to the apical runner, leaf petiole, stem, and shoot apical meristem through the vascular system of the basal runner (Supplementary Fig. S1). Subsequently, we injected *Agrobacterium* solutions (EHA105) into *F. vesca* runners to evaluate GFP reporter expression. CLSM analysis revealed GFP expression in the cortical and epidermal cells of cross-sections at the apical ends of runners injected with *Agrobacterium* carrying the GFP expression vector (Fig. 1F). By contrast, no detectable GFP signal was observed in runners transformed with the pBI121 vector or in control samples without injection (no transfection) (Fig. 1F). These results suggest that *Agrobacterium* injection enables efficient gene expression in transformed vascular tissues, such as strawberry runners. However, when we employed a simplified agroinfiltration method on fully expanded leaves of wild strawberry for further analysis, a higher rate of successful transformation was observed compared to the injection technique.

### Analysis of gene function using leaf infiltration

**Fig. 2:**
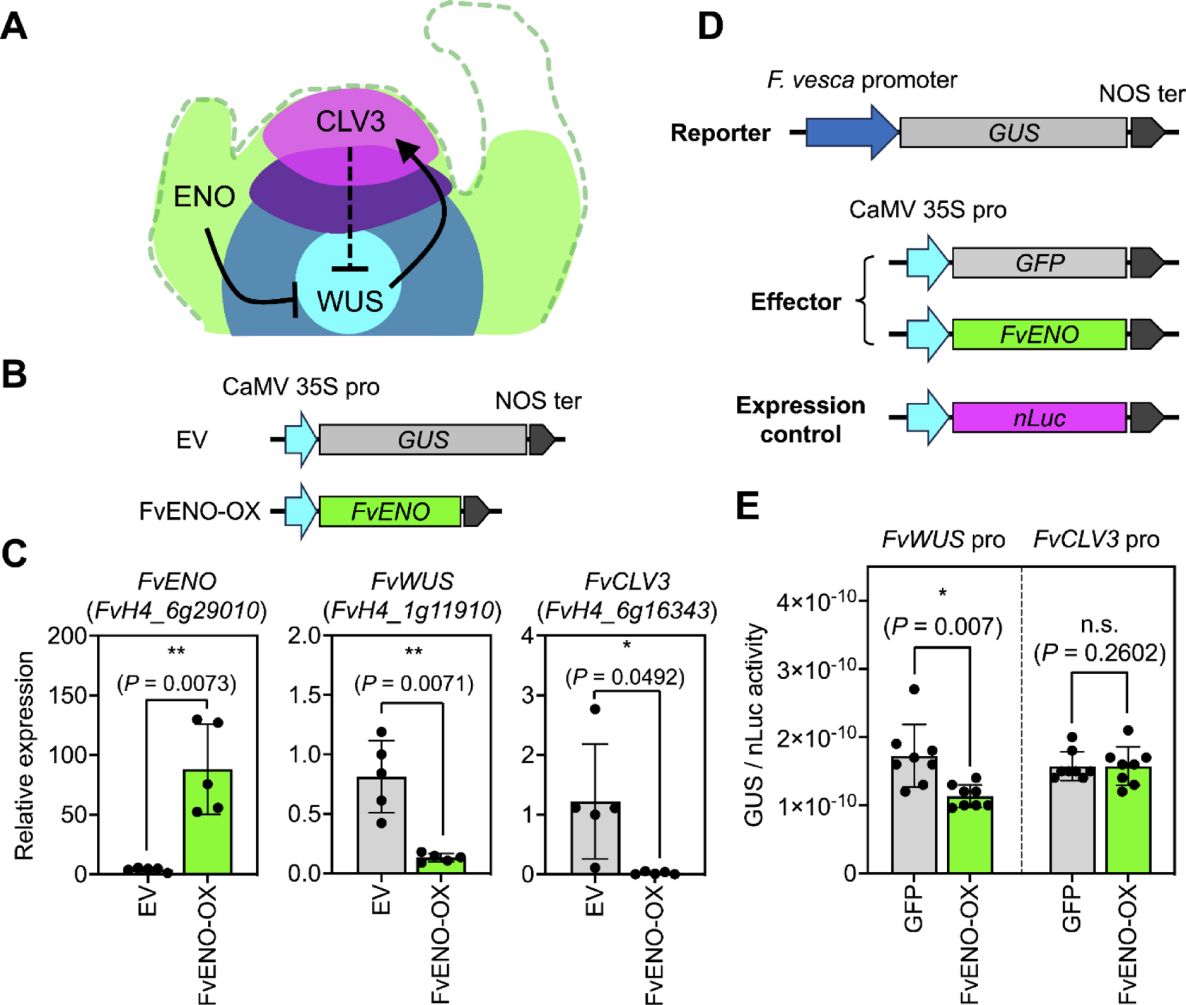
Functional analysis of the FvENO transcription factor using leaf infiltration. (**A**) Regulation of floral meristem development by the ENO/WUS/CLV3 signaling cascade. (**B**) Expression cassettes for transient expression assays of *FvENO* in *F. vesca* leaves using agroinfiltration. EV represents the pBI121 empty vector control. (**C**) *FvENO*, *FvWUS*, and *FvCLV3* transcript levels in agroinfiltrated *F. vesca* leaves at 3 DAI. Error bar represents SD of relative expression levels in five leaves analyzed in different experiments (*n* = 5). Asterisks indicate significant differences in mean relative expression levels analyzed by parametric unpaired *t*-test with Welch’s correction (*; *P* < 0.05, **; *P* < 0.01). (**D**) Promoter-GUS and gene expression constructs used for transactivation/suppression assays of the FvENO transcription factor and its downstream gene promoters in leaves. The nLuc expression vector was used as the control. (**E**) Enzymatic activity of the promoter-GUS reporter in agroinfiltrated *F. vesca* leaves at 3 DAI. Error bar is SD of relative GUS/nLuc activity in eight agroinfiltrated *F. vesca* leaves. Asterisks represent significant differences in mean relative activity analyzed by parametric unpaired *t*-test with Welch’s correction (*; *P* < 0.05, n.s.; no significant difference).

To evaluate the utility of our agroinfiltration method for functional studies of strawberry genes, we conducted a validation focusing on a transcriptional regulation model linked to a commercially important trait in crop plants. The *excessive number of floral organs* (*eno*) locus in the tomato (*Solanum lycopersicum*) genome encodes an AP2/ERF transcription factor known as SlENO. This transcription factor suppresses a regulatory network involved in floral meristem development and fruit size, which includes the tomato *WUSCHEL1* (*SlWUS*; *lc* locus) and *CLAVATA3* (*SlCLV3*; *fas* locus) gene expression pathway (Fig. 2A) ^15,16^. In tomato, SlENO suppresses the transcription of *SlWUS*, resulting in decreased expression of *SlCLV3*. Conversely, the expression of *SlWUS* is also regulated by a negative-feedback mechanism directed by CLV3/CLV1 signal transduction in the apical region of the floral meristem (Fig. 2A). However, the role of ENO in the transcriptional regulation of stem cell signaling mediated by the WUS-CLV3 cascade in strawberry is still unclear.

To confirm the existence of ENO transcription factors and their roles in regulating the WUS-CLV3 signaling pathway in strawberry, we utilized protein sequences from tomato ENO (SlENO), WUSCHEL (SlWUS), and CLAVATA3 (SlCLV3) to identify similar sequences in the genomes of *F. vesca* (Hawaii-4), cultivated strawberries (*F.* × *ananassa*), potato (*Solanum tuberosum*), and *Arabidopsis thaliana* (Table S1). In the *F. vesca* genome, we discovered only one conserved sequence for each protein (Table S1). However, the *F.* × *ananassa* genome contained at least three conserved sequences each for ENO, WUS, and CLV3 (Table S1).

The ENO protein from *F. vesca* shares 50% sequence identity and contains a highly conserved AP2/ERF domain, consistent with ENO transcription factors from other plant species (Supplementary Fig. S2, Table S1). We cloned the coding sequence of the ENO homolog in *F. vesca* (*FvENO*; FvH4_6g29010) and overexpressed it in *F. vesca* leaves using our newly optimized agroinfiltration protocol (Fig. 2B). We analyzed the expression levels of *FvENO* and its putative downstream genes (*FvWUS*; FvH4_1g11910 and *FvCLV3*; FvH4_6g16343) using qRT-PCR. At 3 DAI, *FvENO* transcript levels were significantly elevated in leaves agroinfiltrated with the FvENO expression vector (FvENO-OX) compared to the pBI121 empty vector (EV) control (Fig. 2C). Conversely, *FvWUS* and *FvCLV3* exhibited significantly lower expression levels in FvENO-OX leaves than in EV control leaves (Fig. 2C). Transient expression analysis revealed that FvENO negatively regulates the expression of *FvWUS* and *FvCLV3* in *F. vesca*.

### Transactivation/suppression assays of the strawberry ENO transcription factor

To investigate how FvENO suppresses the transcription of the WUS/CLV3 signaling cascade, we constructed promoter-GUS expression cassettes, introduced them into *F. vesca* leaf cells, and performed transactivation/suppression assays of the FvWUS and FvCLV3 promoter regions by FvENO. We cloned the 2,000-bp DNA sequences upstream of the *FvWUS* and *FvCLV3* coding sequences, including their 5′-untranslated regions, and fused them to the pBI121 expression vector to replace the Cauliflower mosaic virus (CaMV) 35S promoter sequence (Fig. 2D). Subsequently, we introduced *Agrobacterium* cells containing the promoter-GUS expression cassette, the FvENO-OX expression vector, and the nano-luciferase (nLuc) expression control construct into intact *F. vesca* leaves using our newly developed agroinfiltration protocol (Fig. 2D). We analyzed the expression of the GUS and nLuc reporters in agroinfiltrated *F. vesca* leaves at 3 DAI using enzymatic assays. In these assays, GUS reporter activity driven by the FvWUS promoter was significantly lower in FvENO-OX leaves compared to the GFP control (Fig. 2E). However, there were no significant differences in the expression of the *GUS* reporter driven by the FvCLV3 promoter in FvENO-OX leaves compared to the control (Fig. 2E).

### Assessment of promoter activity in wild strawberry

**Fig. 3:**
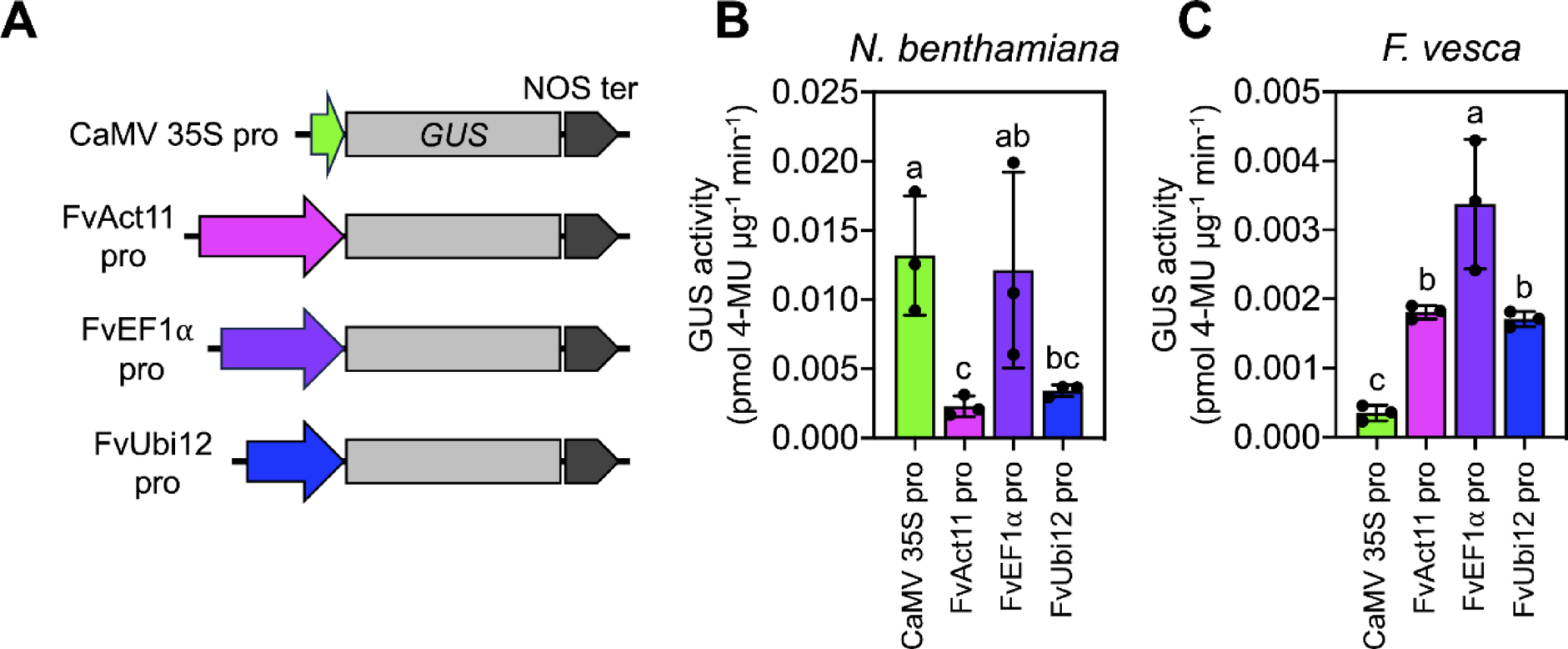
Evaluation of promoters for gene expression analysis in wild strawberry. (**A**) Constructs used to analyze promoter-GUS activity in plants. (**B**) Activities of different promoter-GUS expression cassettes in *N. benthamiana* at 3 days after infiltration. 4-MU, 4-methylumbelliferone. (**C**) GUS reporter activities in transformed *F. vesca* leaves. Error bars show standard deviations of the average GUS activities from three independent experiments (*n* = 3). Different letters indicate significant differences in GUS activity among promoters in each plant species, as analyzed by one-way ANOVA with Tukey’s HSD test at *p* = 0.05.

Although a strong constitutive CaMV 35S promoter is widely used to drive the expression of gene of interest in *F.* × *ananassa* and *F. vesca* ^6,7,11^, we used the leaf infiltration technique to explore alternative constitutive promoters within the wild strawberry genome. We chose the 2.29-kb upstream region of the *F. vesca Actin11* gene (*FvAct11*; FvH4_6g22300), the 1.67-kb promoter segment of *Elongation Factor 1 alpha* (*FvEF1α*; FvH4_3g33150), and the 1.20-kb promoter region of the Ubiquitin C-terminal hydrolase 12-like protein gene (*FvUbi12*; FvH4_1g05490) based on their constitutive expression patterns (Fig. 3A). We fused the native promoter sequences upstream of the *GUS* coding sequence in the pBI121 vector (Fig. 3A) and introduced these vectors into *N. benthamiana* and *F. vesca* leaves through agroinfiltration to compare promoter activities in these two dicot plants.

The GUS activity assays produced distinct results among transfected leaves at 3 DAI. In *N. benthamiana* leaves, the FvEF1α promoter displayed activity comparable to that of the CaMV 35S promoter in regulating the expression of the *GUS* reporter; these activities were significantly higher than those observed using the FvAct11 and FvUbi12 promoters (Fig. 3B). While all native promoters isolated from *F. vesca* led to significantly higher *GUS* expression compared to the CaMV 35S promoter, the FvEF1α promoter demonstrated the greatest activity in inducing *GUS* expression in agroinfiltrated *F. vesca* leaves (Fig. 3C). These results reveal differences in promoter activities in *N. benthamiana* versus *F. vesca*.

## Discussion

Agroinfiltration is a useful tool for elucidating gene function in strawberry ^17^, and extending the agroinfiltration method to intact leaves could offer additional insights into the activities of the gene of interest in biologically active plant cells. The dimorphic leaves of *F. vesca* are typically firm and possess a high cell density ^18^. These properties determine the effectiveness of leaf infiltration protocols and whether transgenes can be expressed at sufficient levels in transfected leaves ^17,19,20^.

Selecting an appropriate *Agrobacterium* strain is critical for achieving optimal transient expression of the target gene in *F. vesca*. Previously, the efficiencies of different strains had not been systematically evaluated. By optimizing co-cultivation conditions and selecting appropriate antibiotic concentrations, GV3101 and LBA4404 achieved high transformation efficiencies of 63% and 67%, respectively, in the stable transformation of *F. vesca* leaf explants ^14^. In this study, in a transient expression assay, *Agrobacterium* strain EHA105 exhibited approximately twice the transformation efficiency in *F. vesca* leaves compared to GV3101 and LBA4404 (Fig. 1). This finding highlights the potential of EHA105 for ensuring the success of optimized agroinfiltration protocols in *F. vesca* and cultivated strawberry species.

The precise regulation of gene expression often relies on the physical interaction between a transcription factor and a *cis*-acting element located in the promoter region of its downstream gene. This transcriptional regulation can be studied using *in planta* transactivation/suppression assays, which involve the transfer of multiple gene expression vectors into leaf cells via *Agrobacterium*-mediated gene transfer ^21^. Transient expression in leaves offers a rapid, accessible, and uniform platform for evaluating gene function in wild strawberry (Fig. 2). Compared to fruit-based agroinfiltration, which is generally limited to later stages of fruit development and often yields inconsistent transformation due to variation in fruit maturity, leaf-based assays provide more consistent results across samples ^6,22^. Additionally, previously reported methods using detached leaves required vacuum infiltration and subsequent regeneration of transformed plantlets that are time-consuming and technically demanding ^5^. In contrast, the method we developed enables direct gene delivery into fully expanded leaves without vacuum or regeneration, streamlining functional assays and making them more suitable for high-throughput analysis.

Disrupting the *SlENO* gene through CRISPR/Cas9-based genome editing resulted in increased expression of *SlWUS* and *SlCLV3* in tomato *eno* mutants ^15,16^. Remarkably, these *eno* mutants exhibited increased numbers of floral organs and locules in fruits, along with a significantly increased fruit size ^16^. Thus, this strategy could readily be used to improve fruit quality traits in strawberries ^23^. Although the protein sequence of FvENO shares only 50% identity with SlENO (Supplementary Fig. 2, Table S1), it is likely that FvENO has a similar function to SlENO, thereby negatively regulating *WUS* and *CLV3* transcription in wild strawberry. Our comprehensive analysis of the transcriptional regulatory mechanism of FvENO highlights the robustness of agroinfiltration of intact leaves for investigating gene function across both wild strawberry (*F. vesca*) and commercially cultivated strawberry (*F.* × *ananassa*) (Fig. 2). The advantages of our agroinfiltration method lie in the gene expression machinery and the uniformity of plant cell types present in intact strawberry leaves ^20^, which enable more nuanced analysis of the proteins governing the mechanisms regulating floral meristem development and fruit size, such as ENO, WUS, CLV3, and other gene products that are abundant in various strawberry tissues. Transient overexpression of FvENO in *F. vesca* leaves led to a reduction in the transcript levels of both *FvWUS* and *FvCLV3*. Although the effects were less pronounced than those observed in the transient suppression assays, the trans-regulation analysis showed that FvENO significantly repressed the activity of the FvWUS promoter, while having no significant impact on the FvCLV3 promoter. This uncoupling between *FvCLV3* transcript repression and promoter activity suggests that FvENO may regulate *FvCLV3* expression indirectly. Given that WUS positively regulates *CLV3* transcription, and that CLV3 peptide, in turn, represses WUS expression mediated by plasma membrane-localized receptor kinase signaling cascade as part of a well-characterized feedback loop, our findings suggest that FvENO may downregulate *FvCLV3* by first repressing *FvWUS* ^16,24^. These results elucidate a potential upstream regulatory role of FvENO in modulating the FvWUS/FvCLV3 signaling axis, which is critical for meristem maintenance and organogenesis.

While WUS and CLV3 are typically expressed in the shoot apical meristem, previous studies have shown that WUS homologs can also be expressed in leaves and floral tissues in certain species, including *F. vesca* ^25,26^. In our experiments, transient overexpression of FvENO in mature *F. vesca* leaves may have induced detectable levels of *FvWUS* transcripts, enabling us to observe its potential regulatory effect on *FvCLV3*. Although leaves are not their primary site of expression, agroinfiltration may create experimental conditions that reveal gene interactions not normally evident ^27^. Further studies are necessary to clarify the exact molecular mechanism by which FvENO influences this pathway, including its spatial and temporal effects on stem cell proliferation and development in *F. vesca* and the cultivated strawberry, *F.* × *ananassa*.

The promoter is a DNA sequence that precisely controls the transcription initiation of its downstream gene, determining whether its expression is enhanced or inhibited ^28^. The distinct regulatory functions of various promoters have been intensively studied in plants, including promoters driving constitutive, tissue- and cell-specific, or inducible gene expression ^29^. The CaMV 35S promoter, a strong constitutive promoter, is widely used to drive the expression of genes of interest in numerous dicot plants, including *F.* × *ananassa* and *F. vesca* ^6,7,11,30^. Although the CaMV 35S promoter induces substantial transgene expression in strawberry, promoters isolated from constitutively expressed strawberry genes may exhibit higher activity and greater efficiency for studying gene function in *F. vesca*.

Our comparative analysis suggests that native promoters have advantages over the CaMV 35S promoter in controlling the expression of target genes in *F. vesca* cells (Fig. 3). This finding is consistent with the results of comparative analyses of the activities of native promoters in other plant species ^31–34^. For example, the oil palm ubi1 promoter exhibited superior activity to the CaMV 35S promoter in driving *GUS* in various oil palm tissues and tobacco leaflets in transient expression experiments ^35^. Moreover, a 650-bp DNA fragment upstream of the strawberry *FaEXP2* gene displayed greater activity than the enhanced 4xCaMV 35S promoter in inducing the expression of the luciferase gene in transformed *F.* × *ananassa* fruits ^36^. The native promoter sequences used in this study, including FvAct11, FvEF1α, and FvUbi12 promoters, represent promising candidates for the overexpression of a gene of interest and as regulatory sequences controlling the expression of guide RNAs and Cas proteins in CRISPR/Cas-genome editing systems in transgenic strawberry lines ^37^. However, the activities of these promoters, whether tissue-specific or constitutive, must be thoroughly examined to optimize their expression patterns and facilitate their extensive application in strawberry.

In summary, we herein developed a practical *Agrobacterium*-based gene transfer technique to investigate the roles of strawberry genes in regulating agronomic traits in intact leaves of wild strawberry (*F. vesca*). This technique sheds light on the regulatory mechanisms potentially controlling floral organ development and fruit enlargement in both wild and cultivated strawberries. Moreover, our optimized agroinfiltration protocol could be used to characterize candidate native promoter sequences isolated from the wild strawberry genome, thereby enhancing the expression efficiency of transgenes such as guide RNA and Cas proteins in CRISPR/Cas gene editing systems for *F. vesca* and *F.* × *ananassa*. This simplified agroinfiltration method represents an important advancement in bridging fundamental research with practical applications. It retains great potential as a versatile tool for functional gene analysis aimed at improving key agronomic traits in strawberry, including pigment biosynthesis, fruit quality, and overall productivity. Incorporating commercial surfactants such as Silwet and Tween-20 might resolve the surface tension of plant cell surface and help *Agrobacterium* penetrate the complex composition of *F. vesca* leaf cells, thereby potentially enhancing transformation efficiency ^4,38^.

## Methods

### Plant cultivation conditions

Seeds of wild strawberry (*Fragaria vesca*, Hawaii-4) were disinfected with 70% (v/v) ethanol in water, soaked in 20% (v/v) kitchen bleach solution (Kao Haiter, Tokyo, Japan), and germinated on 1/2 MS medium + 20 g/l sucrose + 8.0 g/l agar (Sigma Aldrich, Saint Louis, MO, USA). The seedlings were grown at 22 °C under a 12-h-light/12-h-dark photoperiod with 50 µmol photons m^–2^ s^–1^ light in a growth chamber for 4 weeks. Mature plants with approximately four to five fully expanded leaves were transferred from the medium to soil and cultured in a growth chamber under the same cultivation conditions for subsequent transplantation of stolons or for use in gene expression experiments.

For propagation using daughter plants, the stolons were cut approximately 2 cm above and below the daughter plant, and the adaxial part of the plant was placed into premixed soil (Sakata Seed, Kanagawa, Japan). The newly transplanted daughter plants were cultivated under the conditions described above.

*Nicotiana benthamiana* seeds were directly germinated and grown in premixed soil at 25 °C under a 16-h-light/8-h-dark photoperiod using 100 µmol photons m^–2^ s^–1^ light in a growth chamber.

### Plant expression vectors

The plant expression vectors used in this study are listed in Table S2. All expression cassettes used in this study were in the pBI121 backbone. Amplification of DNA fragments for cloning was carried out using PrimeSTAR® GXL DNA Polymerase (Takara Bio Inc., Shiga, Japan). Restricted DNA fragments were then ligated to the linearized pBI121 vector fragments with a Ligation High Ver2.0 DNA Ligation Kit (Toyobo, Osaka, Japan) to construct plant expression vectors. All plasmid DNA molecules were maintained in *Escherichia coli* strain DH5α. The plant expression vectors were isolated from *E. coli* cells using a FastGene Plasmid Mini Kit (Nippon Genetics, Tokyo, Japan).

Total RNA was isolated from fully expanded leaves of *F. vesca* (Hawaii-4) using an RNeasy Plant Mini Kit (Qiagen, Hilden, Germany). The RNA samples were stored at –80 °C. Complementary DNA (cDNA) molecules were synthesized from purified RNA using a ReverTra Ace® qPCR RT Master Mix with the gDNA Remover Kit (Toyobo). Solutions containing cDNA were stored at –30 °C for further experiments. The *GFP(S65T)* gene was cloned using the primers listed in Table S3 and ligated to pBI121 at the *Xba*I/*Sac*I restriction sites. The coding sequence of the ENO transcription factor was cloned from strawberry cDNA samples using the primers listed in Table S3. This sequence was subcloned into pBI121 in the *Xba*I/*Sac*I cloning sites.

The genomic DNA was isolated from frozen *F. vesca* (Hawaii-4) leaf powder using the CTAB DNA extraction/purification protocol ^39^. Promoter regions were amplified from genomic DNA using the primers listed in Table S3. The cloned sequences were ligated to linearized DNA pBI121 at the appropriate restriction sites.

### Transformation of *Agrobacterium tumefaciens* cells

Plant expression vectors were introduced into various *Agrobacterium* strains, including EHA105, LBA4404, MP90, and GV3101, using the freeze–thaw transformation protocol ^40^. Positive transformants confirmed by PCR were used in subsequent transformation experiments.

### *Agrobacterium*-mediated gene transfer

A single colony of *Agrobacterium* carrying the plant expression vector was cultured in LB medium with the appropriate antibiotics at 28 °C and 200 rpm for 18 h. Following incubation, the *Agrobacterium* cells were harvested by centrifugation at 1450 ×g at 4 °C. The cell pellet was resuspended in 5 ml of agroinfiltration solution containing 10 mM MES, pH 5.7, 10 mM MgCl_2_, and 200 µM acetosyringone (Sigma Aldrich). The optical density at 600 nm (OD_600_) of the *Agrobacterium* culture was measured using a spectrophotometer. The *Agrobacterium* culture was adjusted to OD_600_ = 0.2 with agroinfiltration solution and incubated in the dark at room temperature for 2 h. Following incubation, the *Agrobacterium* culture was infiltrated into the abaxial sides of fully expanded leaves in *F. vesca* (Hawaii-4) and *N. benthamiana*. For leaf infiltration in *F. vesca*, the *Agrobacterium* solution was introduced at multiple sites within the central areas between leaf veins (three spots per leaf segment, approximately 30–32 spots per leaf) to ensure thorough dispersion throughout the leaf tissue. Agroinfiltrated leaves were incubated under standard culture conditions for 3 days prior to expression analysis. Plant leaves were rinsed with water prior to sample collection to remove *Agrobacterium* cells from the leaf surface.

### GUS activity assay and histochemical staining

The expression of the bacterial *β*-*glucuronidase* (*GUS*) reporter gene in plants following *Agrobacterium*-mediated transformation was assessed using a GUS activity assay and histochemical staining. For GUS activity assays, individual protein extracts were prepared from liquid nitrogen-ground powder of whole *F. vesca* leaves that had been agroinfiltrated with different *Agrobacterium* solutions, using GUS extraction buffer comprising 50 mM sodium phosphate buffer (pH 7.4), 1.0 mM EDTA (pH 8.0), 10 mM 1,4-dithiothreitol (Sigma Aldrich), 0.1% (w/v) sodium dodecyl sulfate (Sigma Aldrich), 0.1% (v/v) Triton X-100 (Sigma Aldrich), and cOmplete™ Protease Inhibitor Cocktail (Sigma Aldrich). Fifty microliters of total protein solution was incubated with 50 µl of GUS assay solution in a 96-well plate (GUS extraction solution + 1.0 mM 4-methylumbelliferyl *β*-D-glucuronide [Sigma Aldrich]) at 37 °C in a dark chamber for 2–3 h. The catalytic reaction of GUS was terminated by adding 100 µl of 0.2 M Na_2_CO_3_ solution to the well. The fluorescence of the catalytic product 4-methylumbelliferone (4-MU) in the reaction was measured in a microplate reader in fluorescence detection mode with excitation and emission (ex/em) wavelengths of 365 and 455 nm, respectively. The concentration of 4-MU in each sample was determined using the linear regression equation of the fluorescence of standard 4-MU (Sigma Aldrich) prepared at various concentrations.

The concentration of total leaf proteins in the solution was determined using Pierce™ Bradford Plus Protein Assay Reagent (Thermo Scientific, Rockford, IL, USA) with the calibration curve of bovine serum albumin as the standard protein. GUS activity is presented as picomoles of 4-MU per microgram of total proteins per minute (pmol 4-MU µg^–1^ min^–1^).

For GUS staining, agroinfiltrated strawberry leaves were collected and fixed in a fixation solution (10 mM MES, pH 5.7, 0.3% formalin, 0.3 M mannitol) for 30 min. The samples were then washed three times with 50 mM NaH_2_PO_4_ (pH 7.0). GUS staining solution (50 mM NaH_2_PO_4_, pH 7.0; 0.5 mM X-gluc [5-bromo-4-chloro-3-indolyl-*β*-D-glucuronic acid]; 0.5 mM K_3_Fe(CN)_6_; 0.5 mM K_4_Fe(CN)_6_; and 0.01% Triton X-100) was added, and vacuum pressure was applied for 30 min. The samples were then incubated in the staining solution at 37 °C in the dark for 48 h. After staining, the leaves were subsequentially washed with increasing concentration of ethanol solutions (50%, 75%, 90%, and 100%) to remove chlorophyll and dehydrate the tissues prior to observation.

### Nano-luciferase activity assay

Total proteins were extracted from transformed leaves using GUS extraction solution as described above. The activity of the transiently expressed nano-luciferase reporter (nLuc) in the extracts was determined using the Nano-Glo® Luciferase Assay System according to the manufacturer’s protocol (Promega Corporation, Madison, WI, USA).

### Confocal laser-scanning microscopy

The green fluorescent protein (GFP) signal in transformed plant cells was visualized using an SP8X confocal microscope system with a 20 × /0.70 NA immersion objective lens (Leica Microsystems, Tokyo, Japan). A white light laser at 20% intensity was employed for excitation, and a confocal laser-scanning microscopy (CLSM) image was captured at 1024 × 1024 pixels with the 50-pinhole setting. The ex/em wavelengths for GFP were 488/500–550 nm, respectively. Rhodamine B fluorescence in plant tissues was detected with ex/em wavelengths of 560/580–620 nm. The autofluorescence of chlorophyll in the chloroplasts of plant cells was observed at ex/em of 488/680–730 nm. All CLSM images were processed and analyzed using Fiji ImageJ software ^41^.

### Gene expression analysis

Differential gene expression levels in transformed plant cells were assessed using reverse-transcription quantitative PCR (qRT-PCR). cDNA molecules synthesized from RNA samples extracted from leaves as described above, served as a template for qRT-PCR. PCR was conducted with Fast™ SYBR™ Green Master Mix (Thermo Fischer Scientific, Vilnius, Lithuania) and gene-specific primers listed in Table S3. The constitutively expressed housekeeping gene *F. vesca EF1α* (*FvEF1α*) was employed as an expression control. The comparative C_T_ (2^–∆∆C^_T_) method was used to compare gene expression levels in plant tissues ^42^.

### Statistical analysis

The experimental data in this study were obtained from a minimum of three biologically independent samples. Statistically significant differences between two treatments were analyzed using a parametric unpaired *t*-test with Welch’s correction. One-way ANOVA with Tukey’s HSD test at *p* = 0.05 was used to identify significant differences in the means among treatments in an experiment. Statistical analyses were performed, and graphs were constructed using Prism 8.0 (GraphPad Software, Boston, MA, USA).

## Electronic supplementary material

Below is the link to the electronic supplementary material.

Supplementary Material 1

Supplementary Material 2

## Data Availability

The datasets supporting the conclusions of this article are included within the article and as additional files in the supporting information.
