# Supplementary material for: Agroinfiltration technique for elucidating the functions of strawberry genes in Fragaria vesca
Source: Sci Rep. 2025 Jul 1;15:20392. doi: 10.1038/s41598-025-08344-0 (PMC12216212; doi:10.1038/s41598-025-08344-0)
Supplement: Supplementary file 1 — Supplementary Material 1 [file 41598_2025_8344_MOESM1_ESM.pdf]

Supplementary Figures and Tables

Agroinfiltration Techniques for Elucidating the Function of Strawberry Genes in *Fragaria vesca*

Chonprakun Thagun[1] *and Yutaka Kodama*[1, *]

[1] Center for Bioscience Research and Education, Utsunomiya University, Tochigi 321-8505, Japan

Supplementary figure 1
Supplementary table S1 to 3

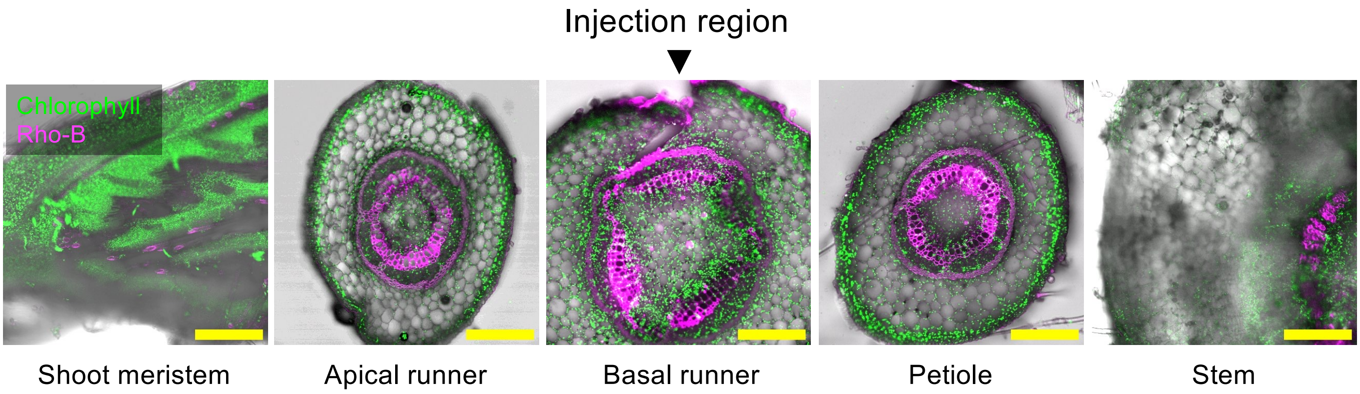

Supplementary figure S1. Fluorescence imaging of rhodomine-B in vascular vessels of strawberry runner.
Translocation of rhodamine-B fluorescence dye (Rho-B) in different vascular tissues of injected strawberry runner was observed using CLSM imaging at 30 minutes post injection. Arrow indicates the injected region of basal segment of runner. Scale bar = 1000 μm.

Table S1. List of plant expression vectors used in this research

Plasmid name	Gene/promoter	Backbone	Restriction sites for insertion	Purpose
pBI121	-	pBI121	-	Control vector
pBI121-GFP(S65T)	*GFP(S65T)*	pBI121	BamHI/SacI	GFP reporter expression
pBI121-FvENO	*FvENO*	pBI121	XbaI/SacI	FvENO expression study and trans-regulation assay
pBI121-FvWUS pro-GUS	*FvWUS pro*	pBI121	HindIII/BamHI	Trans-regulation assay
pBI121-FvCLV3 pro-GUS	*FvCLV3 pro*	pBI121	ScaI/BamHI	Trans-regulation assay
pBI121-FvAct11 pro-GUS	*FvAct11 pro*	pBI121	HindIII/XbaI	Promoter analysis
pBI121-FvEF1α pro-GUS	*FvEF1α pro*	pBI121	HindIII/XbaI	Promoter analysis
pBI121-FvUbi12 pro-GUS	*FvUbi12 pro*	pBI121	HindIII/XbaI	Promoter analysis
pGWB602-nLuc	*nLuc*	pGWB602	-	Expression control

Table S2. List of primers used in this research. Underlined letters present restriction enzyme cutting sites on each primer sequence

Primer name	Primer sequence (5'→3')	Restriction site	Purpose
**Gene/Promoter cloning**			
GFP(S65T)_F	AGGATCCATGGTGAGCAAGGGCGAGG	BamHI	GFP(S65T) cloning
GFP(S65T)_R	AGAGCTCCTACTTGTACAGCTCGTCCATGCC	SacI	
FvENO_F	ATCTAGAATGAGTTTCAAAGCTACCGAGACCG	XbaI	FvENO cloning
FvENO_R	AGAGCTCCTACCAGCTTCCTCTGCAAC	SacI	
FvWUS pro_F	AAAGCTTGAGCGATTATTTGTATCGAATTTCTC	HindIII	FvWUS promoter cloning
FvWUS pro_R	AGGATCCTGGTGATAGTTTTGAGAGAATTGAAGAG	BamHI	
FvCLV3 pro_F	AAGTACTACCGTCGAAATTTCCACGATC	ScaI	FvCLV3 promoter cloning
FvCLV3 pro_R	AGGATCCTGAAGAGCTAGCTTAGTTAAGTGAC	BamHI	
FvAct11 pro_F	AAAGCTTTGAACTCTCTCACCCAGAAAC	HindIII	Cloning of FvAct11 promoter
FvAct11 pro_R	ATCTAGATTTCTATTTGTCTGCTGTCAAAAAC	XbaI	
FvEF1α pro_F	AAAGCTTTACACATTAGCATAACTCTCTTTC	HindIII	Cloning of FvEF1α promoter (partial digestion with HindIII)
FvEF1α pro_R	ATCTAGAGATGAATTAGCTAAATCTGCAATAAG	XbaI	
FvUbi12 pro_F	AAAGCTTACGAAATAATGACAAGTTTGTC	HindIII	Cloning of FvUbi12 promoter
FvUbi12 pro_R	ATCTAGAGTCGTTGAGATCAAACTCG	XbaI	
**qRT-PCR analysis**			
qFvENO_F1	AAACCCGTTTCGTTTCGTTCACC	-	qRT-PCR analysis of gene expression in agroinfiltrated leaf samples
qFvENO_R1	AGGGGGTTCGGTTTTGGAGTC	-	
qFvWUS1_F2	TCCAATCATACCCTTCCTAGCTTCC	-	
qFvWUS1_R2	CAGTCGTGGGAGTCCATCTG	-	
qCLV3-1_F2	ATGGCGTCAAAGTCTATAGCGC	-	
qCLV3-1_R2	AGGATCAGCGCGAGGCTTC	-	
qFvEF1a_F	GGTAACATGATCACACATCCCTTTG	-	
qFvEF1a_R	CTCAGCAGCCTCCTTCTCG	-	

Table S3. BLASTP search of conversed proteins in *Arabidopsis thaliana*, tomato, potato, and strawberries

Protein	E-value	% identity	Bit scores	Gaps
SlENO (Solyc03g117230)				
StENO (Soltu03g031420)	1.04E-116	84	336.7	13
FaFvb6-2-67	2.40E-40	50	143.3	12
FaFvb6-3-157	2.53E-40	49	142.9	12
FvH4_6g29010	2.97E-40	50	142.9	14
FaFvb6-1-161	1.02E-39	50	141.4	14
AT1g80580	2.73E-23	56	97.4	17
SlWUS (Solyc02g083950)				
StWUS (Soltu02g023940)	4.05E-158	89	443.4	15
FvH4_1g11910	2.01E-51	44	172.6	46
FaFvb1-4-57	4.41E-51	44	171.8	46
FaFvb1-3-51	7.22E-49	44	166.0	46
FaFvb1-2-78	3.93E-48	44	164.1	46
AT2g17950	4.98E-37	66	135.2	0
SlCLV3 (Solyc11g071380)				
StCLV3 (Soltu11g027600)	1.89E-59	94	178.7	0
FaFvb6-4-114	1.78E-07	68	47.4	0
FvH4_6g16343	5.71E-07	75	45.8	0
FaFvb6-2-172	7.58E-07	75	45.1	0
FaFvb6-3-315	7.52E-06	71	42.7	0
AT2g27250	1.80E-03	58	37.0	0

AT; *Arabidopsis thaliana*, Fa; *Fragaria × ananassa*, Fv; *Fragaria vesca* (Hawaii-4), Sl; *Solanum lycopersicum*, and St; *Solanum tuberosum*.
